# Strategies towards in vivo imaging of active transglutaminase type 2 using positron emission tomography

**DOI:** 10.1007/s00726-016-2288-y

**Published:** 2016-07-05

**Authors:** Berend van der Wildt, Adriaan A. Lammertsma, Benjamin Drukarch, Albert D. Windhorst

**Affiliations:** 1 Departments of Radiology and Nuclear Medicine, VU University Medical Center, Amsterdam, The Netherlands; 2 Departments of Anatomy and Neurosciences, VU University Medical Center, Amsterdam, The Netherlands

**Keywords:** Transglutaminase type 2, PET, Irreversible TG2 inhibitors, Reversible TG2 inhibitors, Substrates, Antibodies

## Abstract

Transglutaminase type 2 (TG2) is increasingly linked to the pathogenesis of several diseases, such as celiac disease, cancer, and fibrotic and neurodegenerative diseases. In parallel with becoming an attractive target for therapy, interest in the development of compounds for in vivo imaging of TG2 is rising. Such imaging biomarkers might assist in clarifying the role of TG2 in pathology and in monitoring TG2 inhibition in vivo and thus assist in drug development. In this review, the latest results together with various strategies in TG2 PET tracer development are discussed, including radiolabelling of irreversible and reversible active-site inhibitors, as well as allosteric inhibitors, acyl-donor and acyl-acceptor substrates, and anti-TG2 monoclonal antibodies.

## Introduction

**Fig. 1:**
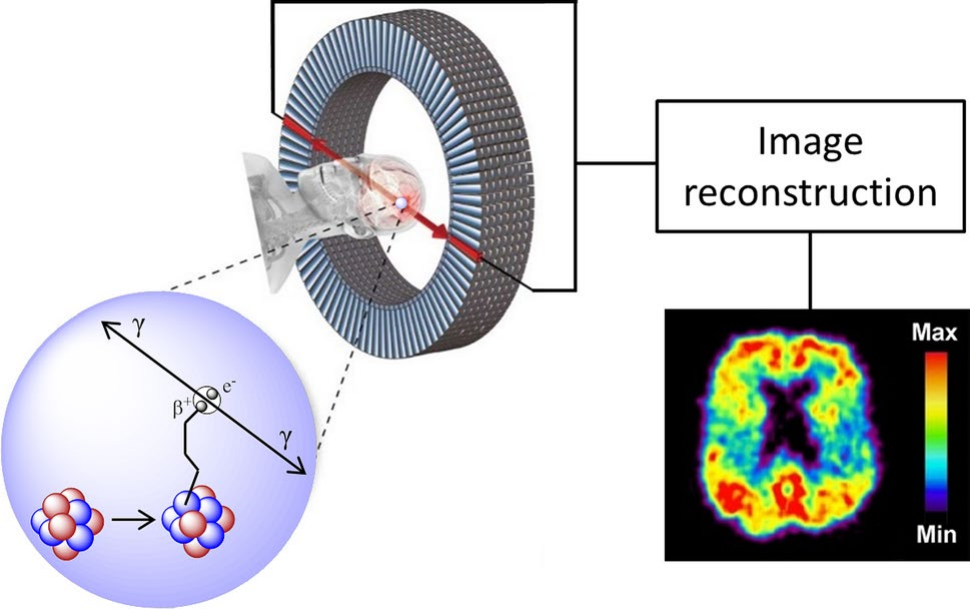
Basic principles of PET. A PET nuclide, incorporated in a biologically relevant molecule, decays and emits a positron inside a subjects’ brain. Following annihilation with a nearby electron (depicted as e^−^), two 511 keV photons are formed, which travel in opposite directions and are detected simultaneously by two opposing (in co-incidence) detector elements within the PET detector ring. Combining co-incidence counts from all detector pairs allows for reconstruction of the quantitative distribution of the radioactive ligand in three-dimensional space

Positron emission tomography is a non-invasive imaging technique that allows for quantitative imaging of biological processes (Phelps 2000; Czernin and Phelps 2002; Bergström et al. 2003; Jones and Rabiner 2012). PET depends on the incorporation of unstable β^+^ emitting nuclides, i.e., nuclides with a neutron deficiency, into biologically relevant molecules. After emission, these positively charged particles, so-called positrons, having a mass equal to that of electrons will collide with electrons in tissue, thereby losing their kinetic energy. Ultimately, a positron will combine with a nearby electron after which the two particles will annihilate, resulting in the emission of two 511 keV photons travelling in opposite direction due to the conservation of angular momentum. Co-incidence detection of these two photons by detectors (often positioned in a circular detector ring), identifies the line of response along which the annihilation took place. Subsequent processing of a large number of such co-incidence events ultimately leads to a three-dimensional map, reflecting the original distribution of the injected radiotracer (Fig. 1). Biologically relevant molecules, in which positron emitting nuclides can be incorporated, include small organic compounds, such as enzyme inhibitors or substrates, receptor agonists or antagonist, and peptides and biologicals, such as monoclonal antibodies and fragments (Miller et al. 2008; van Dongen et al. 2007). The obtained radiopharmaceutical or PET tracer is applied in molecular imaging with PET.

The radiochemistry that is applied to synthesize the PET tracers differs drastically from the traditional organic chemistry in that high sub-stoichiomectric amounts of radioactive reagent, typically in the nanomole range, are reacted with micromolar amounts of the other reagents. The large excess of reagents relative to the small amount of radioactive reagent allows for rapid reactions, compatible with the short radionuclide half-lives. Furthermore, the small scale of the reactions enables rapid purification by means of preparative HPLC. PET tracers are typically obtained in sterile and isotonic injectable solutions and are administered intravenously, allowing for rapid distribution throughout the subject. As a result of the nanomole amounts of tracer compound administered, no biological effects are anticipated, making PET a purely analytical diagnostic imaging modality (Miller et al. 2008). Moreover, PET is a fast and quantitative molecular imaging technique allowing for dynamic molecular imaging and assessment of parameters, such as *B*_max_, *K*_d_, and receptor occupancy, amongst others (Bergström et al. 2003).

For PET imaging using small organic molecules, carbon-11 and fluorine-18 are commonly the nuclides of choice (Miller et al. 2008). Their physical half-lives of 20.3 and 110.8 min are compatible with the in vivo kinetics of such small molecules. Radiosynthesis of these radiotracers is limited by their short half-lives, resulting in limited synthetic toolboxes compared with those in the traditional organic chemistry. Therefore, not all compounds are amenable for radiosynthesis and analogues of those lead compounds may be necessary. In general, monoclonal antibodies do allow for radiolabelling, for example, by modification of lysine residues with a radiometal chelating moiety (Vosjan et al. 2010). Due to their slower kinetics, usually longer lived radionuclides, such as Cu-64, Zr-89, and I-124, are used (van Dongen et al. 2007).

The choice of biologically active compounds for radiolabelling usually is driven by several factors, the location of the biological target being dominant in this selection. Targets located intracellularly or in the central nervous system can only be reached via passive diffusion using small non-polar organic compounds (Pike 2009), whereas extracellular or membrane bound peripheral targets can also be reached using bulkier molecules, such as peptides and antibodies. Furthermore, lead compounds are generally selected based on affinity, selectivity, and metabolic stability (Pike 2009). As PET only measures the distribution of radioactivity and not the chemical form, it is important to carefully study metabolism of a putative radioligand (Pike 2009). At present, the in vivo kinetics of existing TG2 inhibitors which are largely unknown and metabolism data, if available, is largely depending on in vitro plasma or hepatocyte stability data (Wityak et al. 2012; Prime et al. 2012a, b; Badarau et al. 2015). This limited availability of stability data hampers selection of high-potential compounds for translation to PET agents. Radiolabelling of a TG2 inhibitor, however, allows for following its distribution as well as its metabolic stability (Van der Wildt et al. 2016). Furthermore, because of increased sensitivity of LC–MS/MS, formed metabolites may be chemically characterized ex vivo (Ma et al. 2010).

**Fig. 2:**
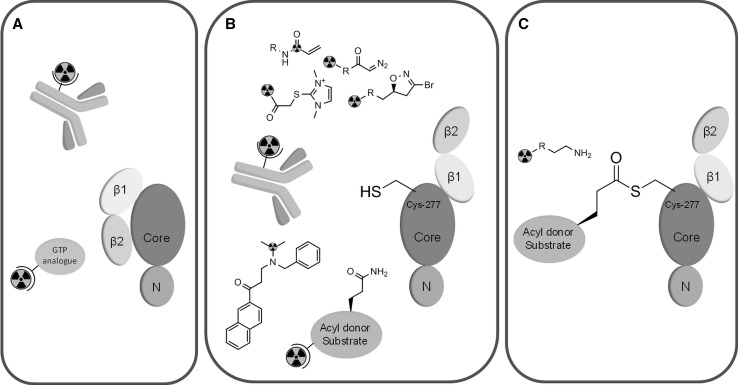
Potential strategies for TG2 PET imaging. **a** Closed conformation TG2 can be targeted by either radiolabelled antibodies or GTP site binding molecules; **b** open conformation TG2 can be targeted by radiolabelled irreversible inhibitors, TG2 antibodies, reversible inhibitors, or acyl-donor substrates; **c** intermediate thioester between acyl-donor substrate and TG2 that can be intercepted by radiolabelled nucleophiles, such as amines

Transglutaminases comprise a class of structurally related enzymes containing eight catalytically active family members, named TG1–TG7 and FXIII (Griffin et al. 2002). These enzymes are well known for their ability of cross-linking proteins by forming an intermolecular isopeptide bond, more specifically an epsilon-(gamma-glutamyl)lysine bond, between the side chains of a glutamine residue as an acyl-donor substrate and a lysine residue of an acyl-acceptor substrate. These isopeptide bonds are highly stable towards proteolysis and introduce mechanical stability in tissues (Griffin et al. 2002). Transglutaminase type 2, TG2, plays an important role during apoptosis by intracellular cross-linking of cellular components, thereby preventing excessive leakage of cellular debris and subsequent inflammatory responses during cell death (Fésüs and Szondy 2005). Extracellularly, TG2 is believed to create a stable environment by forming metabolically stable crosslinks between various matrix proteins (Zemskov et al. 2006). Independent of its cross-linking activity, however, TG2 forms ternary complexes with integrins and fibronectin, resulting in intracellular activation of various processes, thereby assisting in assisting in cell adhesion and migration (Akimov et al. 2000). TG2 cross-linking activity is tightly regulated via various mechanisms. First, TG2 can adapt two distinct conformations (Fig. 2) (Liu et al. 2002; Pinkas et al. 2007). The closed conformation, based on two C-terminal β-barrel domains being tightly folded over the catalytic core domain, does not display cross-linking activity. In this conformation, however, TG2 is known to act as a G-protein involved in signalling processes (Im et al. 1997). The open conformation, where the two β-barrels shift to an upright position parallel to the core and N-terminal domain, results in exposure of the active-site cysteine residue, allowing cross-linking to occur. The latter conformation is induced by high calcium and low GTP/GDP concentrations, whereas the opposite conditions favour the closed conformation. Therefore, it is anticipated that, under physiological conditions, intracellular TG2 has low cross-linking activity, whereas extracellularly, the high calcium and low GTP/GDP concentrations allow TG2 mediated cross-linking. In addition, TG2 cross-linking activity is regulated by the redox state of the enzyme, as a disulfide bond between cysteine 370 and cysteine 371 induces cross-linking inactivation of TG2 (Stamnaes et al. 2010). In addition, by forming ternary protein complexes with integrins and fibronectin, as well as by nitrosylation (Santhanam et al. 2010), TG2 cross-linking activity might be limited (Siegel et al. 2008). Local increases in cross-linking activity of TG2 in vivo may be induced by various molecular stimuli, including retinoids, EGF, and sphingophospholipids (Singh et al. 2001; Antonyak et al. 2009; Lai et al. 1997).

Aberrant TG2 expression and/or activity are increasingly associated with the pathogenesis of a number of disorders, e.g., autoimmune diseases, fibrotic diseases, cancer, and diseases of the central nervous system (Siegel and Khosla 2007). In addition, it has been identified as a target for therapeutic intervention (Pietsch et al. 2013). Presumably best understood is the role of TG2 in celiac disease, where open conformation TG2 mediates the deamidation of dietary gluten peptides via a gluten peptide-TG2 thioester intermediate, resulting in T-cell activation and inflammatory processes (Klöck et al. 2012). In kidney, liver and lung fibrotic diseases, TG2 is suspected to increasingly crosslink extracellular matrix proteins, directly producing scar tissue (Grenard et al. 2001; Johnson et al. 1997; Olsen et al. 2011). In oncology, the role of TG2 is more ambiguous, with either protective anti-apoptotic and pro-apoptotic functions being described (Kotsakis and Griffin 2007). TG2 upregulation has been associated with increased metastasis of various tumour cell lines (Mehta et al. 2004; Satpathy et al. 2007) and drug resistance (Kumar et al. 2010) by promoting epithelial-to-mesenchymal transition, independent of cross-linking activity. In addition, upregulation of the pro-survival transcription factor NF-κB may play a role in drug resistance (Brown 2013), by TG2-mediated cross-linking and deactivation of NF-κB’s endogenous inhibitor IκBα. The TG2 cross-linking dependency remains questionable (Brown 2013). In neurodegenerative diseases, TG2 cross-linking of well-known disease specific proteins, such as amyloid β, huntingtin, and tau, has been associated with the formation of stable neurotoxic aggregates (Wilhelmus et al. 2008).

However, because of its presence in multiple cellular compartments as well as outside the cell (Slife et al. 1985; Lesort et al. 1998), its multiple conformations (Liu et al. 2002; Pinkas et al. 2007), and functions (Gundemir et al. 2012), intrinsically TG2 is a difficult protein to study in vivo. The development of PET tracers for imaging of TG2 activity in vivo may lead to a better understanding of the role of this interesting protein in disease. This review illustrates the potential strategies for TG2 PET imaging and describes potent lead compounds within the context of potential translation to TG2 PET tracers.

## General

**Fig. 3:**
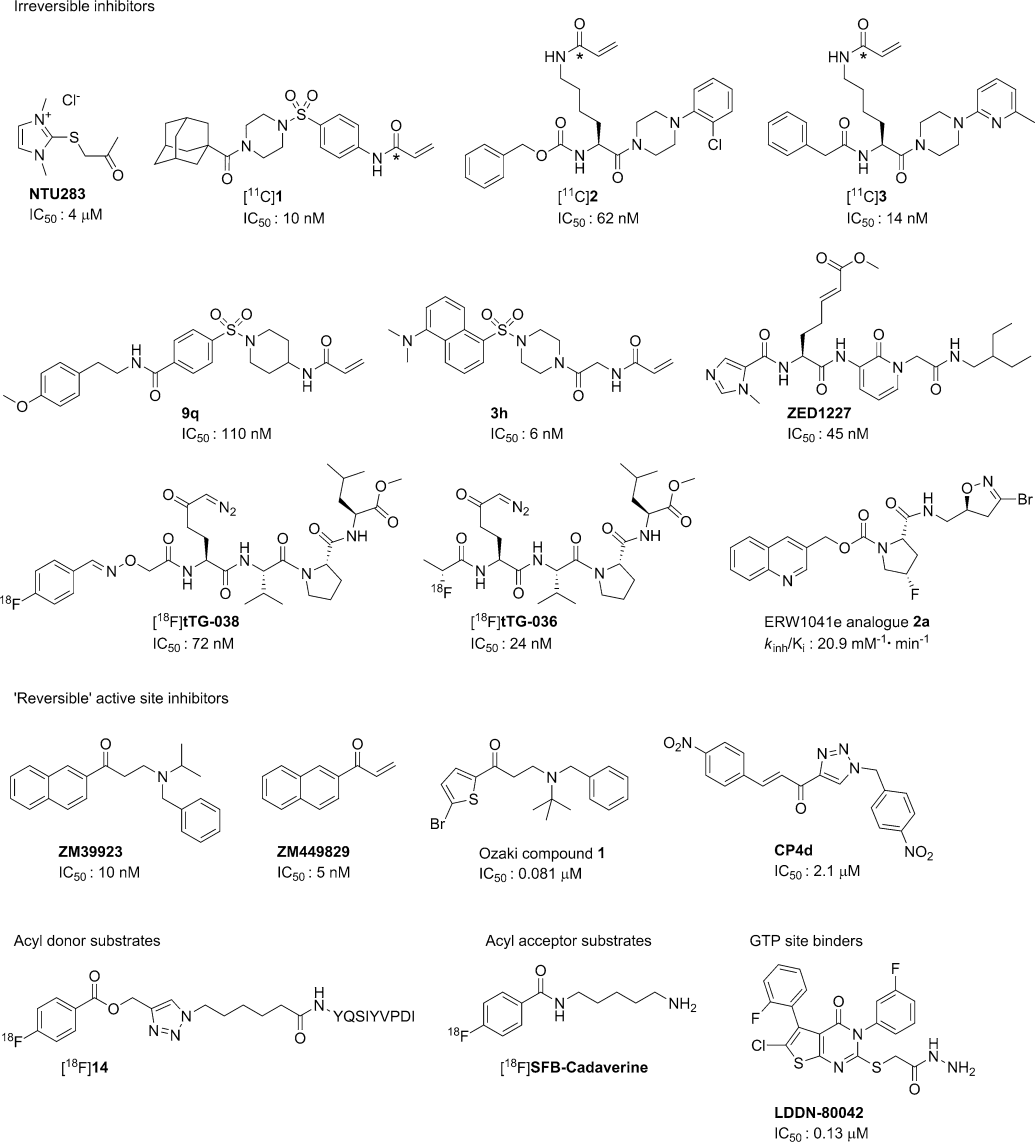
Radiolabelled TG2 inhibitors, substrates, and other compounds that may be suited as lead compounds for the development of TG2 PET imaging agents and as such are discussed in this review. The position of the carbon-11 label in compound [^11^C]**1**–[^11^C]**3** is depicted with *asterisk*

This review will focus on compounds with high potential that justify further elaboration and compounds from different compound classes that allow for different imaging strategies (Figs. 2, 3). As the acyl transfer mechanism of TG2 follows a double displacement reaction (Keillor et al. 2014), various enzymatic states may be intercepted with a PET imaging agent (Fig. 2). In the closed conformation (Fig. 2a), TG2 may be targeted by antibodies or by small organic molecules at the GTP-binding domain. Besides targeting with antibodies, the open conformation (Fig. 2b) may bind to radiolabelled reversible and irreversible TG2 inhibitors, as well as acyl-donor substrates. Finally, the intermediate thioester between TG2 and the acyl-donor may be intercepted by radiolabelled acyl-acceptor substrates (Fig. 2c). Chemical structures of small molecule compounds described in this review are depicted in Fig. 3.

## Irreversible inhibitors

The majority of previously proposed potent TG2 inhibitors are irreversible inhibitors, with recurring moieties, such as diazoketones, α-haloketones, dialkylsulfonium salts, 3-halo-4,5-dihydroxyisoxazoles, and acryl amides or related Michael acceptors (Keillor et al. 2015). The use of PET labelled irreversible TG2 inhibitors should, provided that the newly formed covalent bond is, indeed, irreversible, lead to accumulation of the radiotracer in regions with high-local TG2 cross-linking activity. The first radiolabelled irreversible transglutaminase inhibitor (Freund et al. 1994) was based on the class of imidazole derivatives. By designing two carbon-14 (not a PET isotope) labelled regioisomers of the non-selective inhibitor 1,3-dimethyl-2[(2-oxopropyl)thio]imidazolium chloride (**NTU283** in Fig. 3), the mechanism of transglutaminase inhibition was confirmed as active-site cysteine acetonylation, with the imidazole moiety as leaving group (Freund et al. 1994). Therefore, straightforward carbon-11 methylation on the imidazole moiety of a desmethyl precursor would result in labelling of the leaving imidazole group which would not be retained in tissue, but rather be cleared from the original reaction site. Carbon-11 labelling of the acetonyl functionality is not feasible using the currently available radiochemical toolbox. Analogues of this inhibitor bearing a fluorine-18 atom on the acetonyl group might be developed. **NTU283**, however, suffers from low potency and comparable affinity towards the transglutaminase FXIIIa (Griffin et al. 2008; Freund et al. 1994).

More potent and selective irreversible TG2 inhibitors carrying an acrylamide moiety have recently been reported (**1**–**3** in Fig. 3) (Prime et al. 2012a, b; Wityak et al. 2012). Conveniently, carbon-11 radiosynthesis of acryl amides has been reported using various strategies resulting in the carbon-11 atom at the acrylamide carbonyl position, rendering this class of Michael acceptor inhibitors amenable to radiolabelling (Eriksson et al. 2007). The most convenient amongst these synthetic strategies is the palladium-mediated one-pot [^11^C]CO aminocarbonylation method. In fact, recently, the carbon-11 radiosynthesis together with the evaluation of three potent irreversible TG2 inhibitors radiolabelled using this methodology was described (Van der Wildt et al. 2016). In healthy rodents, however, these compounds displayed low metabolic stability at tracer concentrations, with biological half-lives below 45 min. After selecting the most stable of these three compounds, i.e., compound [^11^C]**3** (Fig. 3), autoradiography experiments on MDA-MB-231 tumour sections, known for its high expression of TG2 (Mehta et al. 2004), demonstrated selective and specific binding to TG2 (Van der Wildt et al. 2016). Nevertheless, TG2-targeting potential of this compound in vivo still remains to be established. Other potent acrylamide inhibitors based on an aminopiperidine core have been reported. Apart from [^11^C]CO aminocarbonylation, the strongest inhibitor within this class, compound **9q**, potentially allows for radiolabelling at the methoxy position by straightforward [^11^C]MeI methylation of the corresponding O-desmethyl precursor (Prime et al. 2012b). Recently, Badarau et al. also reported on the development of potent TG2 inhibitors carrying the acrylamide moiety (Badarau et al. 2015). Unfortunately, the most potent compound **3h**, which is the strongest irreversible inhibitor reported to date, showed low microsomal stability. Compound **3h**, however, did show potency in vivo in a hypertensive nephropathy model, using local administration of **3h** by constant infusion. As for compound **9q**, carbon-11 radiolabelling of **3h** might be performed by aminocarbonylation or *N*-methylation of the *N*-desmethyl precursor. Zedira GmbH has reported the start of Phase I trials using their compound **ZED1227**, a promising peptidomimetic irreversible inhibitor carrying a Michael acceptor other than acrylamide [Zedira GmbH patent filing WO2014012858 (A1), 2014]. This compound is accessible for carbon-11 methylation at either of two methyl groups.

**Z006** (Z-DON-Val-Pro-Leu-OMe) is a highly potent and selective peptidic inhibitor of TG2 (Verhaar et al. 2011; Schaertl et al. 2010). Theoretically, **Z006** allows for radiolabelling at the C-terminal methyl ester employing either [^11^C]MeI or [^11^C]CH_2_N_2_. Methylesters, however, are not very stable in vivo, and, therefore, are rarely the preferred site of introduction of the PET label. Recently, radiosynthesis and preliminary in vivo evaluation of fluorine-18 labelled analogues of **Z006** have been reported ([^18^F]**tTG-036** and [^18^F]**tTG-038** in Fig. 3) (Van der Wildt et al. 2013, 2015). As expected, the methyl ester on [^18^F]**tTG-038** appeared to be metabolically unstable in vivo. Interestingly, however, the resulting carboxylic acid was demonstrated to be metabolically stable and an active TG2 inhibitor as well. Together, these results merit further evaluation of [^18^F]**tTG-038** in vivo. **ERW1041E** (DiRaimondo et al. 2013) was shown to prevent BAP incorporation in a pulmonary hypertension mouse model following intraperitoneal administration. This work concludes that a longitudinal TG2 activity study in PH patients is warranted to confirm TG2 as a biomarker for this disease, emphasizing the necessity for a TG2 PET tracer. To allow radiolabelling, equipotent analogues of **ERW1041E** that are accessible for fluorine-18 labelling were designed as putative future TG2 PET tracers (**ERW1041E** analogue **2a** in Fig. 3). Although **ERW1041E** has shown in vivo inhibition of TG2 activity in this mouse model at 50 mg kg^−1^, it still remains to be elucidated whether this compound class will prove valuable at tracer concentrations. Furthermore, in the previous research, analogues of **ERW1041E** were not able to inhibit TG2 cross-linking activity in an intestinal inflammation model despite various ways of inhibitor administration (Siegel et al. 2008).

## Reversible active-site inhibitors

Binding potential, which reflects *B*_max_/*K*_d_, is a key predictor of the potency of a reversible radiopharmaceutical for imaging a given target [Eckelman and Mathis 2006]. High-affinity compounds are thus preferred, especially if target expression is low. Because little is known about *B*_max_ for TG2 in physiological and diseased states, high-affinity compounds should be selected as starting point for PET tracer development. To date, in contrast to irreversible TG2 inhibitors, only few reversible inhibitors with IC_50_′s in the two digit nanomolar range have been reported. Following screening of an existing drug library, the Janus kinase inhibitor **ZM39923** and its ‘metabolite’ **ZM449829** (Brown et al. 2000; Lai et al. 2008) were reported as potent TG2 inhibitors with 10 and 5 nM IC_50_′s, respectively. Carbon-11 labelling of **ZM39923** should be feasible by a reductive amination reaction between amine precursor and [^11^C]acetone (Berridge et al. 1992) or by alkylation of the corresponding precursor with [^11^C]benzyl iodide (Pekošak et al. 2015). Including 10 mM DTT in the incubation solution, however, resulted in a drastic increase in IC_50_ to micromolar values for both inhibitors (Lai et al. 2008). These IC_50_ values corresponded with those reported in a later study (Schaertl et al. 2010). Although the provided explanation for this phenomenon included, besides an ‘unknown complex mechanism of inhibition’ involvement of these inhibitors in promoting disulfide bonds and thus inactivating the enzyme (Lai et al. 2008), it appears more likely that **ZM39923** is converted to the actual TG2 inhibitor **ZM449829** by a retro Michael reaction (Brown et al. 2000). Using similar assay conditions, i.e., PBS solution, rapid conversion of **ZM39923** to **ZM449829** has already been observed. This newly formed Michael acceptor compound **ZM449829,** in turn, is responsible for irreversible TG2 inhibition. The subsequent cross-reactivity of **ZM449829** towards DTT, as a thiol competing with the active-site cysteine residue, results in a loss of TG2 inhibition. Radiolabelling of **ZM449829** could be achieved on the carbonyl position by a Suzuki or Stille [^11^C]CO insertion reaction (Nader and Oberdorfer 2002). However, the putative cross-reactivity with various thiols will likely limit the chance of successful in vivo TG2 imaging using either [^11^C]**ZM39923** or [^11^C]**ZM449829**. Ozaki et al. also reported on β-aminoketone structures, such as **ZM39923**, as potent active-site TG2 inhibitors (Ozaki et al. 2010). Their most promising compound **1** (Fig. 3) allows for carbon-11 radiolabelling at the *tert*-butyl (Elsinga et al. 1995) or, like **ZM39923**, at the benzyl functionality (Pekošak et al. 2015). It is conceivable, however, that Ozaki compound **1** also acts as a prodrug that via retro Michael reaction converts to an active inhibitor, given the structural similarities to **ZM39923**. No stability data for the developed compounds were provided, nor selectivity data towards other transglutaminases or biological reactive thiols (e.g., glutathione, caspase). In addition, no DTT was used in their assay. Together, it is possible that their reversible inhibitors are actually prodrugs for non-selective irreversible TG2 inhibitors (Ozaki et al. 2010) following a similar mechanism as for compound **ZM449829**. Another class of reported reversible inhibitors is the cinnamoyl-based inhibitors, such as **CP4d** (Fig. 3) (Pardin et al. 2008a, b). Potency of these compounds, however, is limited, hampering their use as lead compound for PET tracer development (Fig. 3, reported IC_50_: 2.1 µM; *K*_*i*_: 174 nM). In addition to the low affinity, although being reported as a reversible inhibitor competitive with the acyl-donor substrate, this compound class could potentially react as irreversible inhibitors. The thiol-reactive Michael acceptor motif is present in the cinnamoyl structure (Esterbauer et al. 1975) and has been shown in related structures to react with biologically active thiols, as present in transglutaminases (Schwöbel et al. 2010). The selectivity data of these compounds towards transglutaminase FXIIIa and cysteineprotease caspase 3, although seemingly promising, are biased by the presence of DTT in these counter assays (Pardin et al. 2008a). DTT, omitted in the TG2 inhibition assay, could very well be acting as a competitive nucleophile for cinnamoyl-based inhibitors and thus preventing FXIIIa and caspase 3 inhibition, suggesting selectivity. The putative irreversible inhibition of these compounds is further encouraged by the fact that modifications at the α,β-unsaturated ketone motif, losing the Michael acceptor reactivity, results in complete loss of potency (Pardin et al. 2008a). Finally, a recent study discussing the reactivity between biological thiols and cinnamoyl-coumarin constructs showed that cinnamoyl compounds with the highest Michael acceptor reactivity carried a *p*-nitrophenyl group, conjugated to the α,β-unsaturated ketone, a structure that is sharing high structural similarities with structure **CP4d** (Aliaga et al. 2014). Therefore, it is highly desirable to evaluate the stability of the inhibitors mentioned above in the presence of thiols and to perform TG2 inhibition experiments in the presence of competing thiols (DTT). These experiments might result in re-evaluation of the exact binding mode of this class of compounds (Pardin et al. 2009).

In summary, it seems that at present, no reversible active-site inhibitors for TG2 with low nanomolar affinities are available. Furthermore, cross-reactivity of irreversible inhibitors, although, perhaps, also for reversible inhibitors, towards thiols (e.g. glutathione, DTT) should be carefully determined prior to starting elaborate SAR studies to avoid false positive in vitro inhibition results.

## GTP-binding site inhibitors

Orthosteric GTP-binding site inhibitors of TG2 as well as inhibitors with unknown binding sites that inhibit GTP-binding have been described (Lai et al. 1998; Duval et al. 2005). Other than GTP itself or closely related analogues, such as dGTP-, GTPγS-, or Bopdipy-modified GTPγS (Datta et al. 2006), no structurally distinct orthosteric inhibitors have been reported, suggesting the GTP-binding site to be highly specialized in this particular binding (Schaertl et al. 2010). In addition, the fact that GTP-binding domains are present in all G-proteins likely discards the GTP-binding site as suitable target for PET radiotracer development, as selectivity issues will arise. In contrast, it has been suggested that the class of hydrazine inhibitors prevents TG2 cross-linking activity by binding allosterically to the GTP-binding site and to multiple enzyme conformers (Duval et al. 2005; Case and Stein 2007; Schaertl et al. 2010). However, due to slow-binding kinetics, sub-optimal affinity, and unknown binding mechanism, PET tracer development based on this hydrazine scaffold appears not to be an attractive strategy.

## Acyl-acceptor substrates

Carbon-14 radiolabelled acyl-acceptor substrates have been at the heart of transglutaminase discovery in the late 1950s, when it was found that liver-derived protein extracts were responsible for amine incorporation into proteins (Sarkar et al. 1957). An important advantage of labelled substrates over inhibitors is the potential signal magnification by means of catabolic trapping, as multiple substrates per transglutaminase enzyme can be incorporated into the local tissue, whereas inhibitors can maximally bind at a 1:1 ratio with the transglutaminase enzyme. Acyl-acceptor substrates for TG2 differ from acyl-donor substrates or active-site inhibitors in that they do not bind to the enzyme itself, but rather compete with naturally present amines, or water, for interception of the intermediate thioester between enzyme and acyl-donor substrate. In contrast to the first step in the cross-linking reaction, the nucleophilic attack on the thioester is not selective (Keillor et al. 2014). In addition, processing of amines in vivo is not limited to TG2, as other transglutaminases as well as other enzymes have amines as substrates. Together, the lack of selectivity of acyl-acceptor substrates will likely hamper the success of this approach towards TG2 PET imaging agents. Nonetheless, TG2-mediated incorporation of the substrate BAP was demonstrated ex vivo in lung tissue using a mouse model of hypoxia-induced pulmonary hypertension (DiRaimondo et al. 2013). In such an ex vivo approach, selectivity data are obtained by pre-treatment of test animals with a TG2 inhibitor after which the free amine substrate is removed by thoroughly washing the tissue sections. In the case of in vivo PET imaging, removal of free ligand by sequential washing procedures is impossible, potentially resulting in a much lower specific signal. Nevertheless, two research groups have reported preliminary results on the fluorine-18 labelling of polyamines, such as putresceine, spermidine, or cadaverine using [^18^F]SFB as amine reactive prosthetic group (Wodtke et al. 2013; Ackermann et al. 2014). In a small pilot experiment using SK-RC-52 tumour bearing mice, a high tumour to blood activity ratio of [^18^F]fluorobenzamide-cadaverine was obtained (Ackermann et al. 2014). Whether this signal was TG2 mediated, however, remains to be elucidated, but it is likely that more in vivo imaging results will be published in the near future.

## Acyl-donor substrates

Although various TG2 specific acyl-donor substrates have been developed for measuring TG2 activity in vitro (Pietsch et al. 2013), to the best of our knowledge, no reports have described ex vivo or in vivo use of radiolabelled acyl-donor substrates for measuring TG2 cross-linking activity. One report has described the radiosynthesis of a fluorine-18 labelled acyl-donor substrate (Vaidyanathan et al. 2009). This fluorine-18 labelled TG2 specific substrate **T32** was selected based on a phage-displayed peptide library screening (Sugimura et al. 2006). Unfortunately, the desired TG2 substrate, [^18^F]**14** was obtained with poor purity and was not formulated in a solution ready for injection. As a consequence, no in vivo work has been reported with this substrate. TG2-reactive peptide sequences should in vivo be incorporated in tissues with a high-local TG2 activity with the same potential signal magnification as described for acyl-acceptor substrates (vide supra). Such peptidic constructs often allow for N-terminal modification to enable chemoselective reaction with a fluorine-18 building block or with a chelating moiety for incorporation of a radiometal of choice. Using a comparable approach, FXIIIa activity imaging has been attempted using ^99m^Tc- and ^111^In-labelled peptidic α2-antiplasmin regions, which were functioning as acyl-donor substrates (Edwards et al. 2006; Nahrendorf et al. 2006). The ^99m^Tc labelled construct showed high uptake in vitro in plasma clots and moderate uptake in vivo in rodent blood clots, which was determined ex vivo by means of a biodistribution experiment. Ex vivo, after thorough washing of the tissues, the ^111^In-labelled construct was shown to be selectively incorporated by FXIIIa in the infarct region in a mouse model of myocardial infarction. Unfortunately, no in vivo imaging was performed in either study. However, it seems likely that a similar strategy, when carefully selecting and developing a high-affinity peptide for TG2, could be used for imaging of extracellular TG2 activity in vivo.

## Antibodies

PET imaging using radiolabelled antibodies, a technique commonly referred to as ‘Immuno-PET’ (Van Dongen et al. 2015), has shown to be well suited for imaging of extracellular targets, such as receptors, and other membrane bound proteins, such as enzymes and transporters. Furthermore, compared to small organic molecules, antibodies generally display less uptake in liver and kidneys and thus allow for PET imaging studies concerning pathologies of these specific organs (Grenard et al. 2001; Johnson et al. 1997). Since TG2 has two highly distinct conformations, the characteristics of the radiolabelled antibody, more precisely. the epitope to which the antibody is directed, will determine what will be measured. When interested in measuring TG2 expression, independent of conformation, the antibody might best be raised against any TG2 domain, though one must consider that extracellularly, the N-terminal domain is likely bound to fibronectin (Hang et al. 2005) and the C-terminal domain involved in integrin binding (Akimov et al. 2000), potentially hampering antibody accessibility. For measuring extracellular TG2 activity rather than expression, the specific antibody should be designed to bind selectively to the open conformation. It must be noted that this is the assumed extracellular conformation due to the mM calcium concentrations in the interstitial fluid (Király et al. 2009). As such, the core domain, including the catalytic cross-linking site, is likely the preferred targeted epitope, as this region is only accessible in the catalytically active open conformation. A TG2 transamidation inhibiting antibody D11D12 has been reported, (patent filing WO2013175229, 2013; Wang et al. 2013), although it has not been reported whether this antibody selectively binds to the open conformation. This TG2 antibody showed inhibition of TG2 transamidation-mediated angiogenesis and wound healing in cellular models (Wang et al., 2013) and further development might result in a huge step towards TG2 transamidation inhibition therapies. Furthermore, the well-known TG2 antibody CUB7402, amongst others, has shown TG2-inhibiting properties by preventing adhesion of Swiss 3T3 fibroblasts (Verderio et al. 1998), although CUB7402 TG2 transamidation inhibiting properties were lost when TG2 was preincubated with Ca^2+^ (Esposito et al. 2002), suggesting that CUB7402 does not bind to the open conformation of TG2, but to TG2 in a closed conformation. It is anticipated that ‘TG2 Immuno-PET’ will be a valuable tool in future research to increase understanding on TG2 biology.

## Future perspectives

With several research groups reporting novel TG2 PET tracers in publications, conference abstracts, and annual reports, we are without any doubt at the frontier of in vivo TG2 PET imaging (Van der Wildt et al. 2013, 2015, 2016; Ackermann et al. 2014; Vaidyanathan et al. 2009), and there is no doubt that results from in vivo TG2 PET imaging studies in animal models of TG2 overexpression/overactivity will appear soon. Furthermore, highly potent small molecule inhibitors are continuously being developed at multiple academic and industrial institutes, increasing the options on lead compound selection for PET radiochemists (Keillor et al. 2015). Table 1 depicts the advantages and disadvantages of potential strategies for TG2 PET radiotracer development.

**Table 1:** *Pro et contra* of TG2 PET tracer development strategies as discussed in this review

Irreversible inhibitors	Reversible inhibitors	GTP-binding site inhibitors	Aryl-acceptor substrates	Acyl-donor substrates	Antibodies
Pro					
High selectivity	High selectivity	Potential imaging of closed TG2	Signal magnification	Signal magnification	Low uptake in kidney and liver
High-affinity	Potentially BBB permeable			Selective	Long circulation
Potentially BBB permeable	Only cross-linking active TG2			Only cross-linking active TG2	High selectivity
Only cross-linking active TG2	Allow for pulse-chase experiments				High-affinity
Contra					
High liver and kidney uptake	Potency sub-optimal	Competing with endogenous GDP	Non-selective	Non-BBB permeable	Expression vs activity
	High liver and kidney uptake	Low potency			Non-BBB permeable
		Independent of cross-linking activity			
		Poor cell penetration			
		Non-selective			

At present, the use of irreversible TG2 inhibitors appears to be an attractive strategy. In particular, irreversible acrylamide inhibitors readily allow for radiolabelling with carbon-11 and can thus be readily evaluated in vivo (Eriksson et al. 2007; Van der Wildt et al. 2016). Labelled acyl-donor substrates, successfully applied for ex vivo measurements of FXIIIa activity (Nahrendorf et al. 2006; Edwards et al. 2006), could also be applicable for imaging of TG2 cross-linking activity. Furthermore, TG2-inhibiting antibodies have been developed that might, unlike small molecules, allow for TG2 imaging in kidney and liver pathologies (Wang et al. 2013). Whether the radiolabelled antibody will reflect local open, closed, or both conformations of TG2 will fully depend on the antibody characteristics. Taken together, it is anticipated that PET will play an important role in moving in vitro and ex vivo biology findings on TG2 into mammalian in vivo studies. As such, it could play a pivotal role in monitoring of target engagement in TG2 drug development.

## Abbreviations

BAP: 5-(Biotinamido)-pentylamine
BBB: Blood brain barrier
B_max_: Maximum binding sites
dGTP: Deoxyguanosine triphosphate
DTT: Dithiothreitol
EGF: Endothelial growth factor
FXIIIa: Activated subunit A of blood coagulation factor XIII
GTP: Guanosine triphosphate
GTPγS: Guanosine 5′-*O*-[gamma-thio]triphosphate
IC_50_: Inhibitor concentration resulting in 50 % inhibition
*K*_d_: Dissociation constant
LC–MS/MS: Liquid chromatography–tandem mass spectrometry
PBS: Phosphate buffered saline
PET: Positron emission tomography
PH: Pulmonary hypertension
SAR: Structure-activity relationship
TG2: Transglutaminase type 2
